# Correlation and underlying brain mechanisms between rapid eye movement sleep behavior disorder and executive functions in Parkinson’s disease: an fNIRS study

**DOI:** 10.3389/fnagi.2023.1290108

**Published:** 2024-01-10

**Authors:** Lu Ren, Xiaoxue Yin, Hai-Yang Wang, Xinqing Hao, Da Wang, Feng Jin, Tingting Zhang, Tao Li, Tingting Zhou, Zhanhua Liang

**Affiliations:** Department of Neurology, First Affiliated Hospital of Dalian Medical University, Dalian, China

**Keywords:** Parkinson’s disease, fNIRS, executive function, rapid eye movement sleep behavior disorder, prefrontal cortex

## Abstract

**Purpose:**

Rapid eye movement sleep behavior disorder (RBD) affects 30%–40% of patients with Parkinson’s disease (PD) and has been linked to a higher risk of cognitive impairment, especially executive dysfunction. The aim of this study was to investigate the brain activation patterns in PD patients with RBD (PD-RBD+) compared to those without RBD (PD-RBD−) and healthy controls (HCs), and to analyze the correlation between changes in cerebral cortex activity and the severity of RBD.

**Methods:**

We recruited 50 PD patients, including 30 PD-RBD+, 20 PD-RBD−, and 20 HCs. We used functional near infrared spectroscopy during a verbal fluency task (VFT-fNIRS) and clinical neuropsychological assessment to explore the correlation between PD-RBD+ and executive function and changes in neural activity.

**Results:**

The VFT-fNIRS analysis revealed a significant reduction in activation among PD-RBD+ patients across multiple channels when compared to both the PD-RBD− and HC groups. Specifically, PD-RBD+ patients exhibited diminished activation in the bilateral dorsolateral prefrontal cortex (DLPFC) and the right ventrolateral prefrontal cortex (VLPFC) relative to their PD-RBD− counterparts. Furthermore, compared to the HC group, PD-RBD+ patients displayed reduced activation specifically in the right DLPFC. Significantly, a noteworthy negative correlation was identified between the average change in oxygenated hemoglobin concentration (ΔHbO_2_) in the right DLPFC of PD-RBD+ patients and the severity of their RBD.

**Conclusion:**

Our study offers compelling evidence that RBD exacerbates cognitive impairment in PD, manifested as executive dysfunction, primarily attributed to reduced prefrontal activation. These aberrations in brain activation may potentially correlate with the severity of RBD.

## Introduction

Parkinson’s disease (PD) is a prevalent neurodegenerative disease characterized by motor and non-motor symptoms (Postuma et al., 2015). Among the non-motor symptoms, rapid eye movement sleep behavior disorder (RBD) is one of the most representative. It is characterized by the absence of skeletal muscle atonia during rapid eye movement (REM) sleep, resulting in dream-related violent movements, swearing, shouting, and other behavioral or emotional reactions (Boeve et al., 2007; Iranzo et al., 2016). Within a decade, about 50% of individuals with idiopathic RBD progress to PD, and ultimately, nearly all (81%–90%) of RBD patients develop a neurodegenerative disorder (Howell and Schenck, 2015). Studies have shown that PD patients with RBD tend to have a longer disease course, more fluctuations in motor symptoms, worse prognosis, and higher incidence of cognitive impairment (Liu et al., 2021; Shrestha et al., 2021; Zimansky et al., 2021). Cognitive impairment, in turn, can reduce the quality of life of PD patients, increase the mortality rate, and elevate the care burden of patients’ families (Lawson et al., 2014).

Numerous studies have reported that PD patients with rapid eye movement sleep behavior disorder (PD-RBD+) are at a higher risk of developing cognitive impairment, experiencing a faster decline in cognitive ability, and having a worse prognosis. RBD has been identified as a potential determinant of dementia development, as evidenced by studies conducted by Schenck et al. (2013), Anang et al. (2014, 2017), and Fereshtehnejad et al. (2015). PD-related cognitive impairment encompasses a range of cognitive domains, including executive function, attention, memory, language, and visual-spatial ability (Kehagia et al., 2010; Litvan et al., 2012). For instance, Naismith et al. (2011) assessed 101 PD patients and found that those with RBD had significantly poorer verbal fluency and working memory than those without RBD (PD-RBD−). Furthermore, a 3-year longitudinal study revealed that PD-RBD+ patients’ executive function, particularly verbal fluency, deteriorated significantly during follow-up (Fereshtehnejad et al., 2015).

At present, the study of cognitive dysfunction, particularly executive function, in PD-RBD+ is still incomplete. A recent study by Figorilli et al. (2020) found a significant decrease in cognitive function in PD-RBD+ patients, particularly in the speech and semantic fluency tests, and also found a positive correlation between RBD frequency and the severity of cognitive dysfunction. However, the lack of control groups represented by PD patients without RBD in this study precludes drawing definitive conclusions. In PD, cognitive impairment often encompasses executive dysfunction and may even precede the onset of motor symptoms (Schapira et al., 2017). Research reveals that individuals with RBD experience cognitive impairments primarily characterized by a decline in executive function (Gagnon et al., 2009). Additionally, the effect of RBD on dementia has been found to mainly occur within the first 5–10 years of PD, and if this time interval is exceeded, the effect may be underestimated (Postuma et al., 2012). Therefore, in the current study, it is necessary to further optimize the experimental design and explore the exact relationship between RBD and cognitive function. Furthermore, a study by Jozwiak et al. (2017) found that male patients with RBD before PD diagnosis had a higher risk of cognitive impairment, highlighting the importance of gender factors in the experimental design. Despite the contradictory findings, studies by Erro et al. (2012) and Rolinski et al. (2014) have shown that there is no significant difference in language fluency test, attention, and visuospatial ability between PD-RBD+ and PD-RBD− patients. The pathogenesis of PD-RBD+ may involve prefrontal cortex dysfunction and abnormal cortical-subcortical functional connectivity, and the potential mechanism of RBD and cognitive impairment in PD remains to be determined. Importantly, prospective studies have shown that the cognitive level of PD-cognitive impairment may have a chance to turn into normal, emphasizing the need to further explore the exact relationship and mechanism between PD-RBD+ and cognitive impairment. This exploration may reveal whether the emergence of RBD is the starting point of faster and more obvious neurodegeneration in the course of PD. Additionally, determining whether specific clinical interventions and neuroprotection are needed for PD-RBD+ patients to delay their disease progression is crucial.

The gold standard for RBD diagnosis is video polysomnography (PSG) monitoring. Nevertheless, the expensive equipment, intricate procedure, high costs, and the demanding nature of the examination can pose challenges for PD patients, who often experience cognitive impairments. Therefore, in clinical RBD screening, the REM Sleep Behavior Disorder Screening Questionnaire (RBDSQ) can serve as a viable alternative (Matar et al., 2021). In a recent extensive longitudinal cohort study, RBDSQ served as the screening tool for RBD, allowing for an investigation into the longitudinal evolution of non-motor symptoms in early-stage PD patients with coexisting RBD (Liu et al., 2021).

Neuroimaging is a valuable tool in understanding the neuropathological mechanisms underlying PD-RBD+. Specifically, it can provide specific biomarkers that may aid in the clinical heterogeneity of PD-RBD+. Functional magnetic resonance imaging (fMRI) has been extensively applied in the study of PD-RBD+, providing evidence that supports our understanding of the brain mechanism underlying this disorder. Studies have shown that PD-RBD+ is associated with prefrontal cortex dysfunction, which has been observed using fMRI (Hanuška et al., 2019; Jia et al., 2022). Moreover, De Micco et al. found that changes in functional connectivity in the neurocognitive network have been detected even in early untreated PD patients without clinically significant cognitive impairment, and these changes are more pronounced in PD-RBD+. These changes in functional connectivity patterns may indicate an increased likelihood of future cognitive dysfunction in PD-RBD+ (De Micco et al., 2023). However, it should be noted that fMRI has high requirements for subject cooperation, and it is difficult to conduct multimodal detection in task states such as movement and language. Most fMRI studies on PD-RBD+ have focused on spontaneous neural activity of the brain in resting state fMRI, and there are few studies on task state fMRI reflecting specific functions. Future studies should aim to address these limitations and incorporate multimodal detection to provide a more comprehensive understanding of the neuropathological mechanisms underlying PD-RBD+.

Functional near infrared spectroscopy (fNIRS) is an emerging brain functional imaging technology that detects changes in cerebral cortex blood oxygen to reflect neuronal activity based on optical principles (Ferrari and Quaresima, 2012; Scholkmann et al., 2014). Compared to other neuroimaging methods, fNIRS has many advantages, including safety, non-invasiveness, anti-motion interference, high comfort, and portability. It has been widely used in the field of neuropsychiatry and has great potential in the study of PD (Ferrari and Quaresima, 2012). In fact, several studies using both fNIRS and fMRI have confirmed the effectiveness and reliability of fNIRS and emphasized its potential in the study of dystonia and other movement disorders (Duan et al., 2012; Pierro et al., 2014; de Faria et al., 2020; Husain et al., 2020). Task-state fNIRS research focuses on inducing neural activity and locating related functional brain regions through specific activation. This method provides a variety of specific detection methods, which are important in evaluating executive function in PD patients. One such method is the classic verbal fluency task (VFT), which has been shown to be effective in assessing executive function (Lang et al., 2021; Wang et al., 2022a). Previously, we employed fNIRS to investigate the cognitive function of PD patients and found that, despite their cognitive assessment scores appearing within the normal range, there was still evidence of impairment in executive function, characterized by reduced activation in different subregions of the prefrontal cortex (Wang et al., 2022b). Importantly, fNIRS can be combined with the VFT to create a paradigm in cognitive neuroscience known as VFT-fNIRS. Notably, fNIRS has advantages over fMRI, which has high subject cooperation requirements and is difficult to use in multimodal detection during task states such as movement and language. While fMRI studies on PD-RBD+ have primarily focused on spontaneous neural activity in the brain during resting state fMRI, fNIRS has the potential to detect specific functional changes during task-state fNIRS. Overall, fNIRS is a promising tool for studying PD and has great potential in advancing our understanding of the neural mechanisms underlying the disease.

Currently, there is a lack of research utilizing fNIRS technology to investigate cognitive function in PD-RBD+ patients. This study seeks to address this gap by comparing the brain activation patterns of PD patients with and without RBD during executive function tasks and by examining the relationship between changes in cerebral cortex activity and the severity of RBD. The overarching goal of this research is to uncover the underlying mechanisms of executive function changes in PD-RBD+ individuals and provide a theoretical foundation for diagnosing cognitive impairments in this patient population.

## Materials and methods

### Participants

Fifty patients diagnosed with primary PD according to the Movement Disorder Society (MDS) criteria in 2015 (Postuma et al., 2015) were recruited for this study. They were divided into PD-RBD+ (*n* = 30, 64.5 ± 7.06 years) and PD-RBD− (*n* = 20, 62.45 ± 9.90 years) groups based on the presence or absence of REM sleep behavior disorder. Twenty healthy controls (HCs) (60.95 ± 6.94 years) were also included (see Table 1 for details). Inclusion criteria for all participants were Pittsburgh Sleep Quality Index (PSQI) score <7, MoCA score ≥26, HAMA score <7, HAMD score <8, and disease duration <10 years. PD patients were further divided into PD-RBD+ (RBDSQ ≥6) and PD-RBD− (RBDSQ <6) groups based on the RBDSQ scores. Exclusion criteria included hearing and dysarthria, other neurological diseases, inability to cooperate for fNIRS assessment, and prior deep brain stimulation surgery. This study was approved by the Ethics Committee of the First Affiliated Hospital of Dalian Medical University (PJ-KS-KY-2021-218) and was conducted in compliance with the Helsinki Declaration. All participants provided written informed consent after understanding the study procedures and potential risks.

**TABLE 1:** Demographic and clinical data between PD with RBD, PD without RBD and HCs.

	PD-RBD+	PD-RBD−	HC	P-value			
	*n* = 30	*n* = 20	*n* = 20	PD-RBD+ vs. PD-RBD− vs. HC	PD-RBD+ vs. PD-RBD−	PD-RBD+ vs. HC	PD-RBD− vs. HC
Age (year)^a^	64.5 ± 7.06	62.45 ± 9.90	60.95 ± 6.94	*P* = 0.29			
Gender (male/female)^b^	15/15	7/13	8/12	*P* = 0.55			
Years of education^a^	11.67 ± 2.80	11.9 ± 3.21	11.9 ± 2.67	*P* = 0.94			
HAMA^a^	3.80 ± 1.00	4.05 ± 1.15	3.5 ± 1.10	*P* = 0.27			
HAMD^a^	3.83 ± 1.29	4.35 ± 1.14	3.85 ± 0.88	*P* = 0.25			
MoCA^a^	27.4 ± 1.33	27.4 ± 1.14	28.15 ± 1.13	*P* = 0.076			
PSQI^a^	6.07 ± 1.17	5.5 ± 1.19	5.55 ± 1.19	*P* = 0.17			
VFT^a^	9.87 ± 4.21	11.65 ± 3.10	12.20 ± 3.29	*P* = 0.068			
RBDSQ^d^	8.93 ± 1.91	0.3 ± 0.47	0.1 ± 0.31	*P* < 0.001	*P* < 0.001	*P* < 0.001	*P* = 0.88
Disease duration (months)^c^	46.27 ± 25.10	39.7 ± 24.82	N		*P* = 0.37		
Hoehn and Yahr stage^c^	1.71 ± 0.46	1.9 ± 0.35	N		*P* = 0.12		
UPDRS-III^c^	27.73 ± 10.11	23.95 ± 7.76	N		*P* = 0.16		
LEDD^c^ (mg/day)	381.1 ± 220.9	355 ± 191.8	N		*P* = 0.67		

PD-RBD+, Parkinson’s disease with RBD; PD-RBD−, Parkinson’s disease without RBD; HC, healthy control; N, not applicable; HAMA, Hamilton Anxiety Scale; HAMD, Hamilton Depression Scale; MoCA, Montreal Cognitive Assessment; PSQI, Pittsburgh Sleep Quality Index; VFT, correct number of words during verbal fluency task; RBDSQ, REM Sleep Behavior Disorder Screening Questionnaire; UPDRS-III, MDS-Unified Parkinson’s Disease Rating Scale-III; LEDD, levodopa equivalent dose.

^*a*^Single-factor-analysis-of-variance (mean ± SD).

^*b*^Chi-square test.

^*c*^Independent sample t-test (mean ± SD).

^*d*^One-way ANOVA and LSD post-test (mean ± SD).

### Data acquisition

#### Clinical, cognitive and sleep assessment

In this study, all PD patients were in the early stage of the disease (Hoehn and Yahr 1.0–2.5) and were evaluated without medication. The MDS-UPDRS III (Goetz et al., 2007) was utilized to evaluate motor symptoms, and the L-dopa equivalent dose (LEDD) was calculated using a standardized formula (Tomlinson et al., 2010). In addition, the severity of anxiety and depression was assessed using HAMA and HAMD, respectively, while cognitive status was evaluated using MoCA. The presence and severity of RBD were assessed using RBDSQ. The PSQI assesses participants’ sleep quality over the past month, suitable for evaluating sleep in those with sleep and mental disorders, as well as for the general assessment of sleep quality. Hence, sleep quality was appraised using the PSQI.

#### Experimental equipment

**FIGURE 1:**
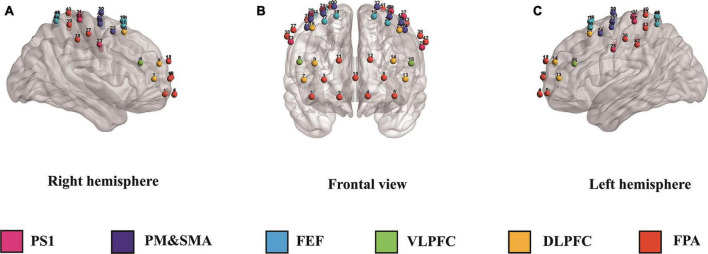
Panels **(A–C)** illustrate the right, frontal, and left hemispheres, respectively, depicting the fNIRS channel layout. PS1, primary somatosensory cortex; PM&SMA, pre-motor and supplementary motor cortex; FEF, frontal eye fields; VLPFC, ventrolateral prefrontal cortex; DLPFC, dorsolateral prefrontal cortex; FPA, frontopolar area.

In this study, we used the Danyang Huichuang portable near-infrared brain function imager NirSmart as the experimental equipment. The device consists of a near-infrared light source emitting at wavelengths of 760 and 850 nm, as well as a receiving detector. The distance between the light source and the probe is 3 cm, and the sampling frequency is 11 HZ. The concentration changes of oxyhemoglobin and deoxyhemoglobin in the cerebral cortex were monitored using the modified Beer-Lambert law. The cap collection is designed based on the 10/20 international standard electrode placement system. For the channel layout, we used 23 transmitting probes and 16 receiving probes that are alternately arranged to form 47 channels. The channel position was determined using a three-dimensional digitizer (FASTRAK, Polhemus, Colchester, VT, USA) through the channel coordinates of the MIN standard template. The specific corresponding brain region was determined based on the distribution probability of the channel between different brain regions. The monitored brain regions included the frontopolar area (FPA), dorsolateral prefrontal cortex (DLPFC), ventrolateral prefrontal cortex (VLPFC), frontal eye fields (FEF), frontopolar area (FPA), and primary somatosensory cortex (PS1) (see Figure 1 and Table 2 for details).

**TABLE 2:** Regions of interest and coordinates of the channels used in the present study.

Brain areas	Left hemisphere	Right hemisphere
Primary somatosensory cortex	CH23, CH28, CH31	CH25, CH37, CH34
Pre-motor and supplementary motor cortex	CH22, CH26, CH29, CH30	CH24, CH32, CH33, CH35
Frontal eye fields	CH17, CH18, CH44, CH45	CH19, CH20, CH46, CH47
Ventrolateral prefrontal cortex	CH8	CH15
Dorsolateral prefrontal cortex	CH7, CH9, CH16	CH13, CH4, CH21
Frontopolar area	CH1, CH2, CH3, CH11, CH27, CH28, CH39, CH40	CH4, CH5, CH6, CH12, CH36, CH41, CH42, CH43
Median frontopolar area	CH10	

#### fNIRS data acquisition

The fNIRS equipment was positioned in a quiet and dimly lit laboratory and preheated before the test to reduce system signal drift and ensure a more stable signal (Haensse et al., 2005). The device was placed behind the subject, and the operator stood at a position to confirm the subject’s line of sight. The subjects sat comfortably in a chair with a backrest that was tilted backward, and a “+” sign was hung on the wall 1 m away in the horizontal direction of the subject’s line of sight. The verbal fluency task (VFT) was used to evaluate executive function. Before the task started, the subjects were instructed to sit comfortably for 5 min, familiarize themselves with the environment, and eliminate tension. During the task, the subjects were instructed to focus on the “+” sign to reduce head or eyeball activity and artifacts. The VFT-fNIRS task consisted of a 30 s baseline period, where the subjects were required to remain still and quiet, followed by the task period, which involved the subjects hearing three Chinese characters, “Jiang,” “Ri,” and “Jia,” with a 20 s interval between each character (Wang et al., 2022b). Subjects were asked to generate as many words as possible within 20 s until the next word was heard. This was followed by another 30 s baseline period. The VFT behavioral data was obtained by recording the total number of correct words spoken by the participants.

#### fNIRS data processing

In this study, we used Matlab (R2013b) and the HomER2 processing program to preprocess the fNIRS data. The primary purpose of preprocessing was to remove noise in the original data and retain the blood oxygen change signal components caused by neural activity as much as possible (Tak and Ye, 2014). We first converted the original intensity data into optical density changes. We then used the spline difference method to detect and correct motion artifacts caused by head movement (Husain et al., 2020). Next, we applied a 0.01∼0.1 Hz band-pass filter to eliminate the influence of physiological and system noise (Strangman et al., 2002). Finally, we transformed the optical density value into the relative concentration changes of averaged HbO_2_ (ΔHbO_2_) and HbR. Because the HbO_2_ signal is more sensitive to regional cerebral blood flow than HbR, we used the averaged ΔHbO_2_ as the hemodynamic response index (Strangman et al., 2002).

We analyzed the averaged ΔHbO_2_ in each patient using the general linear model (GLM). We convoluted the duration of the task with the typical hemodynamic response function, and the baseline was set as 10 s from the end of the pre-task phase and 10 s from the post-task phase. The GLM parameters were estimated in each channel to estimate the activation beta value (β value) under each condition. The task-related β value is the β value in the word formation process minus the baseline β value. We used a single sample *t*-test and two-sample *t*-test to compare the β value within the group and between the two groups. Finally, we visualized the results using BrainNet Viewer (Xia et al., 2013).

### Statistical analysis

Statistical analysis was conducted using SPSS 22.0, and GraphPad Prism 9 was used for data visualization. The experimental data were expressed as standard. In order to evaluate the statistical differences in demographic, clinical variables and behavioral data during VFT tasks among the three groups (see Table 1), appropriate analysis methods were used, and *P* < 0.05 was considered statistically significant. The analysis was initially based on data classification, normal distribution test, and homogeneity of variance test. One-way analysis of variance (ANOVA), independent sample *t*-test, and Chi-square test were used for statistical analyses. Pearson correlation analysis was employed to analyze the correlation between the averaged ΔHbO_2_ and RBDSQ, MoCA, and VFT behavioral counts. In summary, this study employed rigorous statistical methods to analyze the data and ensure the reliability of the results.

## Result

### Demographic and clinical information

A total of 70 participants were included in this study, comprising of 30 patients in the PD-RBD+ group, 20 patients in the PD-RBD− group, and 20 participants in the HC group. There was no significant difference in demographic characteristics such as gender, age, and education level among the three groups. Moreover, there was no significant difference in HAMA, HAMD, MoCA, and PSQI between PD-RBD+ and PD-RBD− groups. However, the RBDSQ scores of PD-RBD+ patients were significantly higher than those of PD-RBD− patients (*P* < 0.001). Furthermore, there was no significant difference in the course of disease, LEDD, and MDS-UPDRS III scores between the two groups (Table 1).

### fNIRS results of VFT task

#### Behavioral results

**FIGURE 2:**
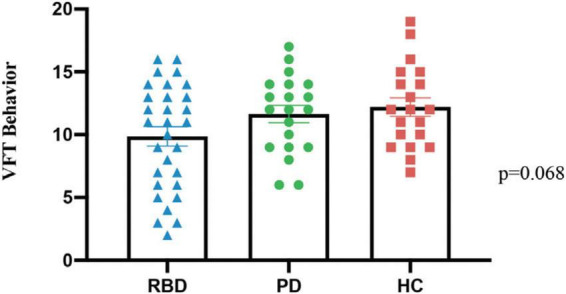
The behavioral data from three groups, PD-RBD+, PD-RBD–, and HC, in the verbal fluency task (VFT).

The number of correct words in the language fluency experiment among the three groups of subjects in the VFT was analyzed using one-way. The results showed that there was no significant difference between the three groups (*P* = 0.068), as shown in Figure 2.

#### Brain activation characterization

Table 3 displays the specific cortical activity changes observed during the VFT task. The outcomes reveal a notable reduction in multi-channel activation for PD-RBD+. Comparative analysis with PD-RBD− indicates a significantly attenuated activation in PD-RBD+ within the right DLPFC (CH 6, 9, and 16; all *P* < 0.05, FDR corrected), left DLPFC (CH 13 and CH 14; all *P* < 0.05, FDR corrected), and right VLPFC (CH 8; *P* < 0.01, FDR corrected). Conversely, in contrast to the HC group, PD-RBD− demonstrates a noteworthy reduction in activation in the right DLPFC (CH 7 and CH 9; all *P* < 0.001, FDR corrected).

**TABLE 3:** Areas with significant changes in cortical activity between groups during VFT task (*P* < 0.05, FDR correction).

	Channel	Side	Brain area	P-value
PD-RBD+ vs. PD-RBD−	CH7, CH9, CH16	Right	DLPFC	0.001–0.034
	CH13, CH14	Left	DLPFC	0.005–0.047
	CH8	Right	VLPFC	0.0056
PD-RBD− vs. HC	CH7, CH9	Right	DLPFC	<0.001

### Correlation analysis

**FIGURE 3:**
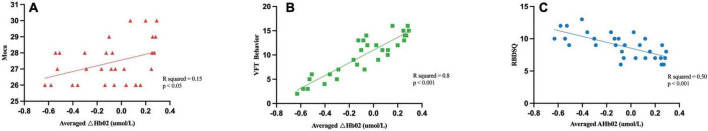
Correlation analysis between the averaged ΔHbO_2_ and demographic and clinical characteristics. The Averaged ΔHbO_2_ of the right DLPFC (CH7) was positively correlated with the MoCA **(A)** and VFT behavior **(B)**, while negatively correlated with the RBDSQ scores **(C)**.

The correlational study revealed that the average ΔHbO_2_ concentration in the right DLPFC (CH7) exhibited positive correlations with MoCA scores (*R*^2^ = 0.15, *P* < 0.05) and VFT behavioral performance (*R*^2^ = 0.80, *P* < 0.001), while demonstrating a negative correlation with RBDSQ scores (*R*^2^ = 0.50, *P* < 0.001). Figure 3 illustrates the results of the correlation analysis between channel activation and clinical data, as well as behavioral performance.

## Discussion

The present study aimed to examine the effects of executive function and spontaneous brain activity on patients with PD-RBD+. To accomplish this objective, we employed functional near-infrared spectroscopy to detect cortical activation during the VFT, and conducted an extensive analysis of the relationship between brain activity changes in PD-RBD+ patients and their demographic, behavioral, and clinical characteristics. It is worth noting that our study is the first attempt to use fNIRS to investigate the underlying brain mechanisms of PD-RBD+ and their connection to executive function.

The results of this study showed no significant differences between PD-RBD+ and PD-RBD− patients in terms of MoCA cognitive measures and VFT-fNIRS behavioral performance. However, PD-RBD+ patients exhibited decreased executive function activation in the bilateral DLPFC and the right VLPFC compared to PD-RBD− patients. The DLPFC is associated with executive function, executive control, decision-making, attention control, working memory, and response selection (Elliott, 2003; Caspers et al., 2017). On the other hand, the VLPFC is involved in spatial attention execution, language, behavior inhibition, and plays a crucial role in response and interference inhibition (Levy and Wagner, 2011; Zhan et al., 2018). DLPFC and VLPFC are integral components of the frontoparietal network (FPN), which are related to working memory, decision-making, goal-oriented behavior, cognitive control, and behavioral inhibition (Levy and Wagner, 2011; Wang et al., 2022b). Previous studies using VFT tasks based on fNIRS have shown that VFT can reflect the executive function of PFC (Zhan et al., 2018). PD cognitive impairment caused by PD-RBD+ is primarily due to executive function damage (Jozwiak et al., 2017; Figorilli et al., 2020). The results of this study suggest a mechanism for decreased FPN activation, including DLPFC and VLPFC, or decreased PD-RBD+ executive function. Many studies have demonstrated that PD patients with RBD are more likely to have cognitive impairment, particularly the progressive deterioration of executive function (Jozwiak et al., 2017; Figorilli et al., 2020). A recent 3-year longitudinal study was consistent with the results of 11 studies and suggested that specific FC dysfunction occurred between DLPFC and PFA during the progression of PD-RBD+, confirming that this change was related to the decline of executive function over time (Jia et al., 2022).

However, recent findings challenge the assumption that cognitive decline is solely related to sleep quality in PD-RBD+ patients. Hermann et al. (2020) conducted a study using the PSQI to evaluate sleep quality in PD-RBD+, PD-RBD−, and HC groups, and found no significant differences in sleep quality between the three groups. Moreover, they observed no significant correlation between cognitive performance and sleep quality in PD patients, further ruling out the possibility that sleep quality is solely responsible for cognitive decline in PD-RBD+ patients. It is worth noting that previous studies have reported conflicting results regarding cognitive performance between PD-RBD+ and PD-RBD− patients (Erro et al., 2012; Rolinski et al., 2014), which could be attributed to differences in sample size, disease duration, age of onset, and cognitive assessment methods. These factors may contribute to the heterogeneity of the study samples and thus impact the results.

Furthermore, studies have demonstrated that VLPFC can regulate wakefulness-related coding during sleep-reward processing, indirectly causing changes in sleep homeostasis during REM sleep (Dolcos et al., 2004). The reduced neural activity of VLPFC in PD-RBD+ patients may be associated with the occurrence of vivid dreams and complex limb movements during the REM phase. Therefore, in the brain activation results of PD-RBD+ patients, the reduction of DLPFC activation may be related to the decline of executive function, while the reduction of VLPFC activation may not only be related to memory and executive function impairment, but also to the occurrence of REM sleep dreams and complex limb movements.

Another significant finding of this study is that the average ΔHbO_2_ in the right DLPFC is negatively correlated with RBDSQ scores, suggesting that changes in brain function mechanism may be related to the clinical manifestations of RBD and significantly reduced executive function changes. This further confirms the potential association between executive function abnormalities in PD-RBD+ patients and DLPFC impairment. Additionally, this study also discovered that, in PD-RBD+, the average ΔHbO_2_ concentration in DLPFC was positively correlated with VFT behavior and MoCA scores, indicating that PD-RBD+ patients have difficulty coordinating cognitive behavior quickly and accurately.

The study findings affirm diminished activation within the FPN, particularly involving the DLPFC and VLPFC in PD-RBD+ patients, closely linked to RBD symptoms. This suggests that the compromised functionality in these brain regions may serve as a potential mechanism underlying sleep disturbances in PD-RBD+ patients. Previous research has highlighted the therapeutic efficacy of neuromodulation techniques, such as repetitive transcranial magnetic stimulation targeting the DLPFC, in treating sleep disorders (Sun et al., 2021) and concurrently enhancing cognitive function in PD patients (He et al., 2021). As a result, the identified brain regions in this study emerge as pivotal targets for neuromodulatory interventions directed at alleviating sleep disturbances and executive function impairments in PD-RBD+ patients.

Based on these findings, it is believed that PD-RBD+ interferes with the prefrontal cortex neural network connection, thereby affecting the role of the DLPFC in cognitive control. Given the inconsistency between the behavioral performance and cognitive assessment of PD-RBD+ patients and the results of brain activation, it is essential to analyze the neural mechanisms that may be related to it. One possible explanation is compensation. Indeed, previous studies have detected differences in functional brain activity between PD patients and control groups without behavioral changes using fNIRS. A recent study found that the neural activity in the right DLPFC of the PD group was significantly reduced during task performance, but there was no difference in behavioral level compared to the control group, indicating the presence of a compensatory mechanism in the brain function of neurodegenerative diseases (Hofmann et al., 2021). Therefore, in this study, the compensatory mechanism may maintain the cognitive ability of PD-RBD+ patients for a certain period.

Several studies have reported that levodopa administration can significantly reduce the spontaneous neural activity in the prefrontal lobe and multiple cerebral cortex areas (Kwak et al., 2012). To avoid the heterogeneity caused by levodopa administration, equivalent doses of madopar were maintained between the PD-RBD+ and PD-RBD− groups in this study. This allowed for the exclusion of drug-induced differences between the groups. Furthermore, it has been reported that the effect of RBD on PD dementia mainly occurs within 5–10 years of the onset of PD (Ferrari and Quaresima, 2012), with this effect possibly being underestimated beyond this period. In this study, patients with a disease duration of less than 10 years were selected, which may explain the existence of compensatory mechanisms despite the changes in brain function networks. However, longitudinal studies are necessary to further verify these findings and explore the potential differences in cognitive impairment progression between PD-RBD+ and PD-nRBD groups. While the VFT behavioral data demonstrated lower scores in PD-RBD+ patients when compared to the HC group, the observed differences did not achieve statistical significance. This potential decline in executive function warrants further validation through studies involving a larger sample size. However, no significant cognitive scores difference was observed between PD-RBD+ and PD-RBD− groups, suggesting that PD-RBD+ patients exhibit executive impairment at a neurological level even when cognitive function is relatively normal.

The experimental design of this study is the first to explore the correlation between PD-RBD+ and cognitive impairment and the specific brain mechanisms underlying these changes using fNIRS equipment. The results have important clinical implications, as studies have demonstrated that neural networks are plastic and PD mild cognitive impairment may still have the opportunity to return to normal levels. There is currently no specific intervention for PD-RBD+ cognitive impairment, which can progress rapidly and increase the risk of developing dementia, adversely affecting the quality of life of patients and their families (Nomura et al., 2013). Therefore, this study may provide a theoretical basis for early intervention in PD-RBD+ and increase the likelihood of favorable outcomes for patients with PD-RBD+ mild cognitive impairment.

There are several limitations to this study that must be acknowledged. Firstly, the relatively small sample size used in this preliminary and exploratory study necessitates that the findings be validated and extended through larger-scale investigations, as well as the implementation of longitudinal research on PD-RBD+. Secondly, despite the administration of levodopa being controlled, and patients refraining from taking PD drugs for at least 12 h prior to data collection, the impact of long-term drug accumulation and short-term drug residuals on cerebral cortex function could not be entirely excluded. Thirdly, while the RBDSQ is a widely employed screening tool for RBD in this study, it is important to acknowledge that its accuracy and reliability in patient inclusion may be influenced in comparison to the gold standard for RBD diagnosis, which is video PSG (Nomura et al., 2011). Additionally, sleep apnea was not monitored using PSG in this study, and considering the potential impact of sleep apnea on cognitive function, the interpretation of results in this study should be approached with caution. Future research endeavors should contemplate the integration of video PSG to thoroughly assess the potential influence of sleep apnea on study outcomes, and further enhance research reliability. Lastly, as a cross-sectional study, this research could not determine the progression of cognitive function and neural activity changes over time in PD-RBD+ patients.

## Conclusion

We utilized fNIRS brain functional imaging to investigate the relationship between brain mechanisms and executive function in PD-RBD+ patients. Firstly, the results of the VFT-fNIRS study indicated that the activation of the FPN, including the DLPFC and VLPFC, was reduced, suggesting a mechanism for the decline in executive function in PD-RBD+ patients. Secondly, we observed a negative correlation between the average ΔHbO_2_ in the right DLPFC and RBDSQ scores, indicating an association between RBD and diminished activation in the mentioned brain region. These findings offer potential targets for further neuromodulatory interventions, such as repetitive transcranial magnetic stimulation.

## Data availability statement

The original contributions presented in this study are included in this article/supplementary material, further inquiries can be directed to the corresponding authors.

## Ethics statement

The studies involving humans were approved by the Ethics Committee of the First Hospital of Dalian Medical University. The studies were conducted in accordance with the local legislation and institutional requirements. The participants provided their written informed consent to participate in this study.

## Author contributions

LR: Writing – review & editing, Writing – original draft, Conceptualization, Data curation, Formal analysis, Investigation, Methodology, Project administration, Software, Visualization. H-YW: Writing – review & editing, Conceptualization, Data curation, Formal analysis, Investigation, Methodology, Project administration, Software, Supervision, Visualization, Writing – original draft. XY: Writing – original draft. XH: Writing – original draft. DW: Writing – original draft. FJ: Writing – original draft. TZa: Writing – original draft. TL: Writing – original draft. ZL: Project administration, Funding acquisition, Investigation, Resources, Supervision, Writing – review & editing. TZo: Writing – review & editing, Project administration, Funding acquisition.
